# Patients with Dilated Cardiomyopathy and Sustained Monomorphic Ventricular Tachycardia Show Up-Regulation of *KCNN3* and *KCNJ2* Genes and *CACNG8*-Linked Left Ventricular Dysfunction

**DOI:** 10.1371/journal.pone.0145518

**Published:** 2015-12-28

**Authors:** Ana Ortega, Estefanía Tarazón, Esther Roselló-Lletí, Carolina Gil-Cayuela, Francisca Lago, Jose-Ramón González-Juanatey, Juan Cinca, Esther Jorge, Luis Martínez-Dolz, Manuel Portolés, Miguel Rivera

**Affiliations:** 1 Cardiocirculatory Unit, Health Research Institute of La Fe University Hospital (IIS La Fe), Valencia, Spain; 2 Cellular and Molecular Cardiology Research Unit, Department of Cardiology and Institute of Biomedical Research, University Clinical Hospital, Santiago de Compostela, Spain; 3 Cardiology Service of Santa Creu i Sant Pau Hospital, Barcelona, Spain; 4 Heart Failure and Transplantation Unit, Cardiology Department, La Fe University Hospital, Valencia, Spain; Indiana University, UNITED STATES

## Abstract

**Aims:**

Disruptions in cardiac ion channels have shown to influence the impaired cardiac contraction in heart failure. We sought to determine the altered gene expression profile of this category in dilated cardiomyopathy (DCM) patients and relate the altered gene expression with the clinical signs present in our patients, such as ventricular dysfunction and sustained monomorphic ventricular tachycardia (SMVT).

**Methods and Results:**

Left ventricular (LV) tissue samples were used in RNA-sequencing technique to elucidate the transcriptomic changes of 13 DCM patients compared to controls (n = 10). We analyzed the differential gene expression of cardiac ion channels, and we found a total of 34 altered genes. We found that the calcium channel CACNG8 mRNA and protein levels were down-regulated and highly and inversely related with LV ejection fraction (LVEF) (r = –0.78, *P*<0.01). Furthermore, the potassium channels KCNN3 and KCNJ2 mRNA and protein levels were up-regulated and showed also a significant and inverse correlation with LVEF (r = –0.61, *P*<0.05; r = –0.60, *P*<0.05) in patients with SMVT.

**Conclusion:**

A broad set of deregulated genes have been identified by RNA-sequencing technique. The relationship of *CACNG8*, *KCNN3* and *KCNJ2* with LVEF, and the up-regulation of *KCNN3* and *KCNJ2* in all patients with SMVT, irrespective of *CACNG8* expression, suggest a significant role for these three ion flux related genes in the LV dysfunction present in this cardiomyopathy and an important relationship between *KCNN3* and *KCNJ2* up-regulation and the presence of SMVT.

## Introduction

Dilated cardiomyopathy (DCM) is one of the most common cardiomyopathy prevalent worldwide; and also one of the main causes of cardiac transplantation. This disease causes heart failure (HF) and is characterized by ventricular dysfunction, chamber enlargement and abnormal left ventricular (LV) wall thickness [1]. Despite the studies analyzing the development of HF, the fundamental mechanisms responsible for its onset and progression remain only partially understood [2].

HF has been associated with heart alterations at different levels [3–6]. Cardiac ion channels are the key components required for the proper contraction of heart muscle, and their disruptions have been described in HF [7, 8], which evidences their importance in this syndrome. We previously carried out a study throughout microarray technology to identify the differentially expressed ion channel genes in DCM patients [5]. The results of this earlier study formed the basis for further investigation in this area, more works are necessary to fully understand the role of cardiac ion channels at genetic level to serve as future therapeutic application.

Next-generation sequencing technologies have facilitated the investigation of entire mammalian transcriptomes [9]. Among these approaches, RNA-sequencing (RNA-seq) is a powerful, feasible and affordable technology that provides accurate transcriptome analysis. Compared to microarrays, RNA-seq generates novel and valuable information for the characterization of a high variety of diseases, at an unprecedented depth and sensitivity.

In the present study, RNA-seq technique was used to evaluate human HF transcriptome changes in the ion channel category, comparing LV tissue samples of DCM patients with controls (CNTs). We sought to investigate the relationship of the expression profile of these ion flux related genes with the overall LV depression and with the presence of sustained monomorphic ventricular tachycardia (SMVT) in some of our patients.

## Methods

### Source of tissue

LV samples were obtained from 13 DCM patients undergoing cardiac transplantation and 10 non-diseased donor hearts as CNT samples. The clinical history, ECG, Doppler echocardiography, hemodynamic studies, and coronary angiography data were available on patients. Non-ischemic DCM was diagnosed when patients had intact coronary arteries on coronary angiography, and LV systolic dysfunction (LV ejection fraction [LVEF] <40%) with a dilated non-hypertrophic left ventricle (LV end-diastolic diameter [LVEDD] >55mm). Twenty three per cent of the DCM patients were diagnosed with SMVT and 10 out of 13 were free of this arrhythmia. To improve the numerical base with a higher number of patients in protein analysis we increased the DCM samples up to 19. All patients were functionally classified according to the New York Heart Association (NYHA) criteria and received medical treatment according to the guidelines of the European Society of Cardiology [10].

The CNT hearts were initially considered for cardiac transplantation donation, but were subsequently deemed unsuitable either because of blood type or size incompatibility. The causes of death in the CNT group were cerebrovascular or motor vehicle accidents. All hearts had normal LV function and no history of myocardial disease at the time of transplantation.

The project was approved by the Ethics Committee (Biomedical Investigation Ethics Committee of La Fe University Hospital of Valencia, Spain) and was conformed in accordance with the principles outlined in the Declaration of Helsinki [11]. All heart samples were obtained with written informed consent of patients.

The LV samples were collected from near the apex of the left ventricle and maintained in 0.9% NaCl at 4°C for a maximum of 6 h after the coronary circulation loss, and then stored at –80°C until RNA extraction and protein determination. The appropriate handling and the rapid sample collection and storage by our on call (24 hours) team, lead to these high quality samples (RNA Integrity Number (RIN) >9 in all samples). The sample’s handling was carried out equally in both groups.

### RNA isolation

The tissue samples were homogenized using TRIzol® agent in TissueLyser LT (Qiagen; UK). RNA was extracted using the PureLink™ Kit (Ambion Life Technologies; CA, USA), following the manufacturer’s recommendations. The RNA concentration was measured on the Nanodrop 1000 spectrophotometer (Thermo Fisher Scientific; UK), and the purity and integrity of RNA samples were measured using the microfluidics-based platform 2100 Bioanalyzer with the RNA 6000 Nano LabChip Kit (Agilent Technologies; Spain). All RNA samples displayed a 260/280 absorbance ratio ≥2.0 and reached a minimal RIN >9.

### RNA-seq analysis and data computational analysis

The description of RNA-seq procedure and data computational analysis are extensively described by Rosello-Lleti et al [12]. The data presented in this paper have been deposited in NCBI’s Gene Expression Omnibus (GEO) [13] and are accessible through GEO Series accession number GSE55296 (http://www.ncbi.nlm.nih.gov/geo/query/acc.cgi?acc=GSE55296).

### RT-qPCR analysis

Reverse transcription was carried out using 1 μg total RNA and M-MLV enzyme (Invitrogen Ltd, UK) according to the manufacturer’s protocol. The resulting cDNA was used as a template for RT-qPCR in a high throughput thermocycler (ViiA^™^ 7 Real-Time RT-PCR System, Applied Biosystems, Foster City, CA, USA) according to the manufacturer’s instructions using the following TaqMan® probes: *KCNN3* (Hs01546821_m1), *KCNJ2* (Hs00265315_m1). The housekeeping genes *GAPDH* (Hs99999905_m1), *TFRC* (Hs00951083_m1) and *PGK1* (Hs99999906_m1) were used as endogenous controls.

### Protein analysis

The materials and protocols used for the homogenization of samples, protein determination, polyacrylamide gel electrophoresis, Western blot analysis and fluorescence microscopy analysis are described in detail by Ortega et al [14].

Protein samples for detection of CACNG8, KCNN3 and KCNJ2 were separated using Bis-Tris Midi gel electrophoresis with 4–12% polyacrylamide. The following antibodies were used: anti-CACNG8 rabbit polyclonal (ab116142) (1/1000), anti-KCNN3 rabbit polyclonal (ab28631) (1/500), anti-Kir2.1 (KCNJ2) (1/1000) rabbit monoclonal (ab109750), and anti-GAPDH mouse monoclonal (ab9484) (1/1000) as a loading control.

### Statistical methods

Data were expressed as the mean ± standard deviation for continuous variables and as percentage values for discrete variables. The Kolmogorov–Smirnov test was applied for analyzing the data distribution. Clinical characteristics of patients were compared by using Student’s t-test for continuous variables and Fisher’s exact test for discrete variables. Significant mean differences between groups with a normal distribution were analyzed by using the Student’s t-test, and the nonparametric Mann–Whitney *U* test was performed for comparisons between data that were non-normally-distributed. The *KCNMB3* mRNA levels exhibited a non-normal distribution and were log transformed (and proved to be normalized) before parametric correlation analysis. Finally, Pearson’s correlation coefficients were calculated to determine the relationships among variables. *P* < 0.05 was considered statistically significant. All statistical analysis was performed using the SPSS software (version 20.0) for Windows (IBM SPSS Inc; Chicago. IL, USA).

## Results

### Clinical characteristics of patients

We analyzed 13 human hearts from patients diagnosed with DCM and 10 non-diseased donor hearts used as CNT samples in RNA-seq technique, and 19 samples of DCM patients and 10 CNT for protein analysis. DCM patients in both studies were mainly men (92% and 84%, respectively) with a mean age of 51 ± 11 and 47 ± 12 years, respectively. The patients had an NYHA functional classification of III–IV and were previously diagnosed with significant comorbidities. Table 1 shows the clinical characteristics of the DCM patients. The CNT group was also mainly composed of males (80%), with a similar mean age of 47 ± 16 years.

**Table 1:** Clinical characteristics of dilated cardiomyopathy (DCM) patients.

	DCM (n = 13)	DCM (n = 19)
	RNA-sequencing	Western Blot
Age (years)	51 ± 11	47 ± 12
Gender male (%)	92	84
NYHA class	3.4 ± 0.4	3.5 ± 0.4
BMI (kg/m^2^)	27 ± 5	25 ± 5
BNP (pg/mL)	8529 ± 11577	6025 ± 11818
Hemoglobin (mg/dL)	13 ± 3	13 ± 3
Hematocrit (%)	39 ± 8	40 ± 6
Total cholesterol (mg/dL)	147 ± 37	145 ± 44
Prior hypertension (%)	17	29
Prior smoking (%)	50	67
Diabetes mellitus (%)	17	13
Heart rhythm sinusal (%)	69%	74%
EF (%)	20 ± 7	22 ± 8
FS (%)	11 ± 4	12 ± 4
LVESD (mm)	71 ± 12	66 ± 12
LVEDD (mm)	80 ± 11	75 ± 12
Left ventricle mass index (g/m^2^)	241 ± 77	208 ± 72
Duration of disease (months)	75 ± 68	80 ± 69

DCM, dilated cardiomyopathy; NYHA, New York Heart Association; BMI, body mass index; EF, ejection fraction; FS, fractional shortening; LVESD, left ventricular end-systolic diameter; LVEDD, left ventricular end-diastolic diameter.

### Gene expression analysis of RNA-seq data and RT-qPCR validation

We performed a large-scale RNA-seq study to identify novel genes affecting the development and progression of DCM. This analysis identified 2398 differentially expressed genes between the DCM and CNT groups (≥1.3–fold, *P* < 0.05). We compared the differentially expressed genes identified by both RNA-seq and microarray techniques and found 209 altered genes common to both analyses, and 2189 differentially expressed genes identified only by the RNA-seq analysis.

Among the deregulated genes of the high-throughput analysis, we found that some belonged to the cardiac ion channel category. Thirty-four genes altered in the DCM group were involved in ion fluxes, of which 16 were up-regulated and 18 down-regulated (Table 2). Of these genes, we found those that were implicated in this category studied and validated in the previous microarray experiment (*CACNB2*, *SCN2B*, *KCNJ5*, *CLIC2* and *CLCN3*).

**Table 2:** Cardiac ion channel genes differentially expressed in dilated cardiomyopathy (DCM) patients.

		DCM	
Gene symbol	Description	Fold Change	p-value
*CACNB2*	calcium channel, voltage-dependent, beta 2 subunit	-1.60953	0.033
*CACNG8*	calcium channel, voltage-dependent, gamma subunit 8	-2.46702	0.033
*CLCN3*	chloride channel, voltage-sensitive 3	-1.51139	0.020
*CLIC2*	chloride intracellular channel 2	-1.56718	0.014
*CLIC3*	chloride intracellular channel 3	2.08832	0.019
*CLIC5*	chloride intracellular channel 5	-1,6376	0.049
*HCN3*	hyperpolarization activated cyclic nucleotide-gated potassium channel 3	1.81734	0.031
*HCN4*	hyperpolarization activated cyclic nucleotide-gated potassium channel 4	-2.07491	0.002
*KCNA4*	potassium voltage-gated channel, shaker-related subfamily, member 4	1.36218	0.034
*KCNA6*	potassium voltage-gated channel, shaker-related subfamily, member 6	2.93070	0.006
*KCNAB1*	potassium voltage-gated channel, shaker-related subfamily, beta member 1	1.90995	0.011
*KCNC1*	potassium voltage-gated channel, Shaw-related subfamily, member 1	-1.64	0.047
*KCNC3*	potassium voltage-gated channel, Shaw-related subfamily, member 3	2.05616	4.04E-05
*KCNC4*	potassium voltage-gated channel, Shaw-related subfamily, member 4	1.75287	0.001
*KCND3*	potassium voltage-gated channel, Shal-related subfamily, member 3	-1.38738	0.005
*KCNE3*	potassium voltage-gated channel, Isk-related family, member 3	2.20996	0.001
*KCNE4*	potassium voltage-gated channel, Isk-related family, member 4	-1.53151	0.026
*KCNH8*	potassium voltage-gated channel, subfamily H (eag-related), member 8	-2.13393	0.006
*KCNIP2*	Kv channel interacting protein 2	-2.35017	0.002
*KCNJ12*	potassium inwardly-rectifying channel, subfamily J, member 12	-1.59201	0.001
*KCNJ2*	potassium inwardly-rectifying channel, subfamily J, member 2	1.61034	0.006
*KCNJ3*	potassium inwardly-rectifying channel, subfamily J, member 3	3.20796	0.013
*KCNJ4*	potassium inwardly-rectifying channel, subfamily J, member 4	1.79831	0.019
*KCNJ5*	potassium inwardly-rectifying channel, subfamily J, member 5	-1.66220	0.034
*KCNK1*	potassium channel, subfamily K, member 1	-1.95354	0.008
*KCNMB3*	potassium large conductance calcium-activated channel, subfamily M beta member 3	-1.68	0.014
*KCNN2*	potassium intermediate/small conductance calcium-activated channel, subfamily N, member 2	-1.53786	0.014
*KCNN3*	potassium intermediate/small conductance calcium-activated channel, subfamily N, member 3	2.02008	0.005
*P2RX6*	purinergic receptor P2X, ligand-gated ion channel, 6	1.87630	0.021
*SCN1A*	sodium channel, voltage-gated, type I, alpha subunit	-1.95057	0.021
*SCN2B*	sodium channel, voltage-gated, type II, beta subunit	2.10081	7.10E-05
*SCN3A*	sodium channel, voltage-gated, type III, alpha subunit	2.08531	0.005
*SCN3B*	sodium channel, voltage-gated, type III, beta subunit	1.77089	0.020
*TRPM7*	transient receptor potential cation channel, subfamily M, member 7	-1.38567	0.006

**Fig 1:**
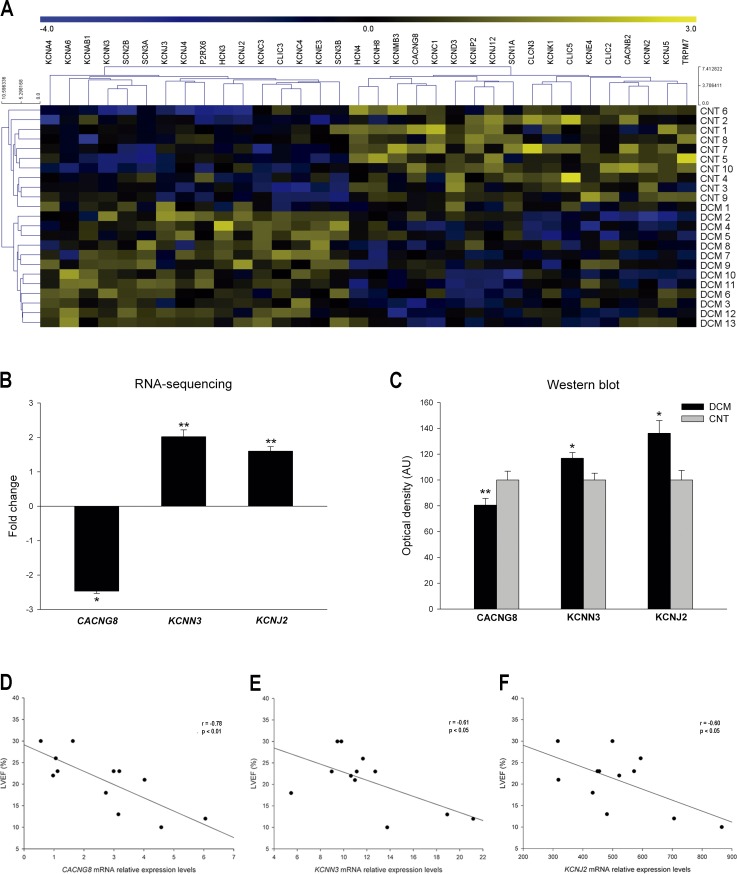
Alterations in cardiac ion channel expression in human DCM patients. (**A**) Heat map with hierarchical clustering of the transcriptomic analysis. (**B**) mRNA expression levels of *CACNG8*, *KCNN3* and *KCNJ2* in DCM patients compared to CNT subjects. (**C**) Protein levels of CACNG8, KCNN3 and KCNJ2 in DCM patients. The values of the CNT group in the analysis of mRNA expression (**B**) were set to 1 and in the western blot were set to 100 (**C**) and were previously normalized to GAPDH. The data are expressed as mean ± standard error of the mean of the fold change levels and in optical density AU in the western blot. *P<0.05, **P<0.01, vs. the CNT group. (**D, E and F**) Relationship of *CACNG8*, *KCNN3* and *KCNJ2* with LVEF. The relative levels are calculated based on data normalization by the initial genome read counts per sample or per library size [12].

We performed a Heat map and hierarchical clustering in which the two groups, DCM and CNT, show a clear separation in two different gene expression profiles (Fig 1A). We focused our study on the only three ion channel genes found related with LV function. The calcium channel gene *CACNG8* showed a down-regulation in the DCM subjects (-2.46 fold, *P*<0.05). In contrast, the potassium channels *KCNN3* and *KCNJ2* showed increased mRNA levels (2.02 fold, *P*<0.01 and 1.61 fold, *P*<0.01, respectively) (Fig 1B) in all DCM patients. All the patients with SMVT showed a *KCNN3* and *KCNJ2* up-regulation. RT-qPCR analysis validated the potassium mRNA levels (2.41 fold, *P*<0.05 and 1.81 fold, *P*<0.05, respectively).

### Western blot and immunofluorescence analysis

Protein levels of CACNG8 calcium channel (80±23 vs. 100±21 arbitrary units [AU], *P*<0.01) and KCNN3 and KCNJ2 potassium channel proteins (117±17 vs. 100±16 AU, *P*<0.05; 136±44 vs. 100±20 AU, *P*<0.05, respectively) were all in accordance with the previously measured mRNA levels (Fig 1C). Fluorescence images showed a clear distribution of KCNJ2 protein in the plasma membrane and different fluorescence intensity between DCM and CNT group (143±23 vs. 100±31 AU, *P*<0.01).

### Relationship of cardiac ion channel genes with LV dysfunction

We determined the relationship between the differentially expressed ion channel genes and the ventricular dysfunction of patients. In 12 of the 13 DCM samples, the LVEF was available. The calcium channel gene expression levels showed a very good relationship with LVEF (r = -0.78, *P*<0.01) (Fig 1D), although no relationship was found with the presence of SMVT in a particular individual.

We also found good relationships between LVEF and potassium channels mRNA levels (r = -0.61, *P*<0.05 and r = -0.60, *P*<0.05, respectively) (Fig 1E and 1F). Of the 34 ion flux related genes analyzed, these three were the only altered genes that were found significantly related to LVEF, and two of them were up-regulated in all patients diagnosed with SMVT.

## Discussion

DCM is one of the most frequent pathologies causing HF. In this disease, impairment in proper cardiac contraction occurs [1]. This process is carried out by ion currents whose concentration changes at different stages of action potentials to produce contraction and relaxation of cardiac fibers [15]. Cardiac ion channels are thus the key components of this fine–tuned process, and their deregulation can cause heart dysfunction.

We performed an RNA-seq technique to elucidate and compare the differentially expressed genes in DCM patients. Among these genes, we identified genes included in the ion channel category. RNA-seq allowed identification of a higher number of differentially expressed genes (2189 additional altered genes) and also genes codifying ion channels (21 additional altered genes) as compared to the microarray technique, providing a greater depth and sensitivity level in addition to have revealed consistent results among datasets.

Three out of the 34 identified ion channel deregulated genes have shown a relationship with LV function, being important in cardiac dysfunction. Previous studies have shown that *CACNG8* encodes a subunit of L-type calcium channels, whose are directly implicated in cardiac contraction. CACNG8 is involved in regulating the gating properties and maintaining an inactive state of the channel [16]. This action could be reduced when the subunit is down-regulated, as we show in this study, provoking a prolonged opened state of the pore-forming subunit. Our results show a strong inverse relationship between *CACNG8* expression and the depressed LV function, and most importantly, we identify *CACNG8* as the only calcium-flux-related gene linked to ventricular function. Therefore, down-regulation of *CACNG8* gene and protein may be due to a regulation mechanism aimed at restoring the contraction–relaxation function in DCM, being its relationship with LV function in accordance with this hypothesis.

*KCNN3* encodes a small conductance Ca^+2^- activated K^+^ channel which is responsible for I_KCa_ currents and is activated by intracellular Ca^+2^ ions [17, 18]. Modulates cardiac repolarization and it has been reported to have an important role in ventricular tachycardia [19]. However, there are some controversies about its specific function in rhythm alterations, while some authors report a protective role when the I_KCa_ current is blocked [19, 20], or a pro-arrhythmic role when the gene is overexpressed [21], evidencing a negative effect of this current, others have shown that a blockage of this current promotes the development of ventricular arrhythmias [22, 23]. In this work, we show an up-regulation of *KCNN3* which contributes to an increase in I_KCa_ current, these findings are consistent with other studies in which an up-regulation of this ion channel in HF is reported [24, 25] and, in addition, we report an inverse relationship with LV function. These findings suggest that in human DCM the up-regulation of *KCNN3* may be contributing to a worsening of ventricular function and the up-regulation found in all patients with SMVT could indicate that this gene may participate in the induction of ventricular tachycardias.

*KCNJ2* is responsible for K^+^ inward rectifier current (I_K1_) charged to cardiomyocyte initial depolarization and final repolarization. Increases of its expression have been related with an increase in I_K1_ current [26]. This ion channel has been implicated in several rhythm disturbances. Concretely, mutations of gain of function are related to a shortening of action potential duration in atrial fibrillation (AF) [27] and also to short QT syndrome [28, 29], both characterized by ventricular tachycardia induction. Also it has been shown an increase of this channel in DCM patients [30]. Consistent with these studies, we have found an up-regulation of this gene in our DCM patients, and an inverse relationship with LV function, suggesting that KCNJ2 elevated levels promote an I_K1_ current increase that leads to ventricular tachycardia and affect also the mechanical function of the heart.

Here, we show that both potassium genes, previously related to human arrhythmias, are up-regulated in all DCM patients with SMVT, which lead us to conclude that changes in mRNA levels play an important role in the onset of these rhythm disturbances.

Another interesting gene that has shown an alteration in this study in DCM patients is *KCNIP2* which, together with the other studied genes *KCND3* and *KCNA4*, contributes to I_to_ current [31, 32], being important determinants of ventricular repolarization [33]. Some authors report that the loss of I_to_ current in *KCNIP2*-deficient mice is not a triggering event of HF, considering this down-regulation a consequence of the disease [34]. *KCNIP2* and *KCND3* are down-regulated in HF, as reported by several authors in animal models and humans [32, 35–38], and *KCNA4* is up-regulated [32], being our findings in this work in concordance with these previous data. The different expression tendencies observed in these genes responsible for I_to_ current, suggest an expression modulation of regulatory subunits for a cooperative functioning aimed at maintaining the proper potassium current of the ion flux in DCM end-stage patients.

Therefore, the relationship of *CACNG8*, *KCNN3* and *KCNJ2* with LVEF, and the up-regulation of *KCNN3* and *KCNJ2* in all patients with SMVT, irrespective of *CACNG8* expression, suggests a significant role for these three ion flux related genes in the LV dysfunction present in this cardiomyopathy and an important relationship between *KCNN3* and *KCNJ2* up-regulation and the presence of SMVT.

### Study limitations

Our study was conducted in DCM patients with no existence of family history of DCM. Nevertheless, a common limitation in studies using human samples is the pharmacological treatment that could influence our results. Moreover, our tissue samples are confined to transmural left ventricle apex, so our findings could not be generalized to all regions of the left ventricle. However, our work was performed using a suitable sample size of both patients and CNTs.
